# Multiple applications of metal-organic frameworks (MOFs) in the treatment of orthopedic diseases

**DOI:** 10.3389/fbioe.2024.1448010

**Published:** 2024-09-04

**Authors:** Ziwen Zhao, Chenxu Wang, Aiguo Liu, Ning Bai, Bo Jiang, Yuanfu Mao, Ting Ying, Daming Dong, Chengqing Yi, Dejian Li

**Affiliations:** ^1^ Department of Orthopedics, The First Affiliated Hospital of Harbin Medical University, Harbin, China; ^2^ Department of Orthopedics, Shanghai Pudong Hospital, Fudan University Pudong Medical Center, Shanghai, China; ^3^ Department of Orthopedics, The First Affiliated Hospital of Henan University, Kaifeng, China; ^4^ Department of Gastroenterology, Huaihe Hospital of Henan University, Kaifeng, China; ^5^ The First Affiliated Hospital of Ningbo University, Ningbo, China; ^6^ Shanghai YangZhi Rehabilitation Hospital (Shanghai Sunshine Rehabilitation Center), School of Medicine, Tongji University, Shanghai, China

**Keywords:** metal-organic frameworks, MOFs, tumor, osteoporosis, osteoarthritis

## Abstract

Pharmacologic treatment of orthopedic diseases is a common challenge for clinical orthopedic surgeons, and as an important step in the stepwise treatment of orthopedic diseases, it is often difficult to achieve satisfactory results with existing pharmacologic treatments. Therefore, it is increasingly important to find new ways to effectively improve the treatment pattern of orthopedic diseases as well as to enhance the therapeutic efficacy. It has been found that metal-organic frameworks (MOFs) possess the advantages of high specific surface area, high porosity, chemical stability, tunability of structure and biocompatibility. Therefore, MOFs are expected to improve the conventional traditional treatment modality for bone diseases. This manuscript reviewed the applications of MOFs in the treatment of common clinical bone diseases and look forward to its future development.

## 1 Introduction

As the foundation of the human body structure, bones have important functions such as supporting the body, protecting organs, supporting movement and blood production. Orthopedic diseases usually accompany people throughout their lives, can occur at different ages. With the aging of the global population and the increase in the popularity of sports, there has been a significant increase in the proportion of orthopedic disorders in the clinical setting. These diseases are often caused by trauma, infection, inflammation, degenerative changes, and oxidative stress, resulting in bone-related diseases such as bone fractures, osteoarthritis, bone tumors, and osteoporosis, which pose a serious threat to human health. However, in the face of the increasing number and complexity of orthopedic diseases, conventional drug therapy has considerable limitations in clinical application, such as the lack of controlled-release properties of drugs, weak targeting ability, and easy degradation of many drugs during the delivery process, resulting in unsatisfactory therapeutic effects, so the conventional drugs in the treatment of orthopedic diseases have entered a bottleneck. Therefore, there is an urgent need to find more efficient and safer drug treatments.

With the development and advancement of nanomedicine, the mechanical, biological and electrochemical properties of nanomaterials have brought many nanomaterials into the field of researchers’ vision and achieved good results. Nanomaterials are characterized by ultra-thin thickness and layers of precise chemical functional groups and the thickness of 2D nanomaterials is even as small as 0.34 nm, which is equivalent to the distance between adjacent bases of DNA (Li et al., 2001). Common clinical nanomaterials such as polycaprolactone (PCL), polylactic-co-glycolic acid (PLGA), nanohydroxyapatite (nHA), zirconium oxide nanoparticles (nZr), silicon dioxide nanoparticles (nSi), silver nanoparticles (AgNPs), graphene oxide (GO), MOFs, etc (Bharadwaz and Jayasuriya, 2020). Nanomaterials can be categorized by shape and size. For example, inorganic nanoparticles, organic polymer based nanoparticles and extracellular vesicles belong to zero-dimensional nanomaterials, which can be used for drug delivery (Wang et al., 2021), imaging (Kim et al., 2018) and therapy (Kim et al., 2020; Kwon et al., 2016; Bjorge et al., 2017). GO has been sought after by many researchers in recent years, it always been used as a molecular carrier and an enhancer of cell attachment (Liu et al., 2013; Lee et al., 2011). Its decorated polymer scaffolds can be used for tissue regeneration (Yadid et al., 2019). Among numerous nanomaterials, MOFs stands out in recent years and attracts the attention of doctors and researchers.

**FIGURE 1:**
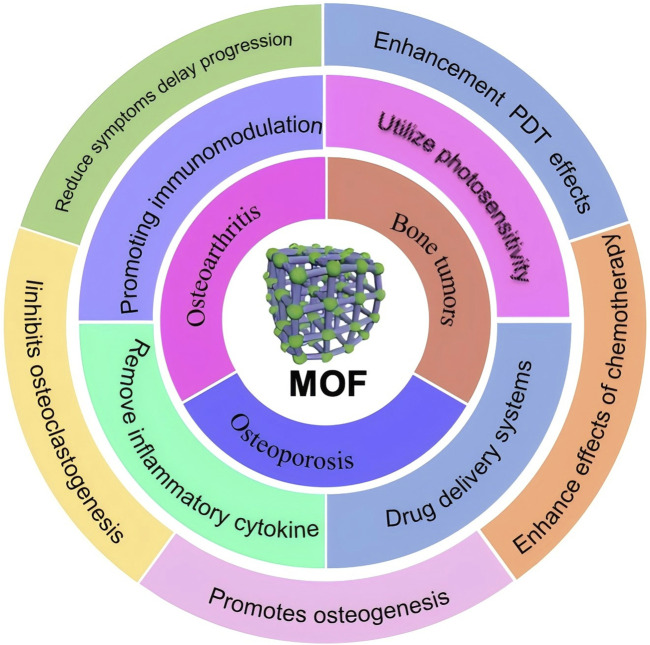
Schematic illustration of application of MOF in different orthopaedic diseases in this review.

In 1995, Yaghi et al. (1995) reported in the journal Nature a coordination compound with a two-dimensional structure synthesized from a rigid organic ligand trimesic acid and transition metal Co., referred to it as MOF. In 2004, Ferey et al. (2004); Ferey et al. (2005) reported two sieve type MOFs with ultra large pore characteristics, MIL-100 and MIL-101. They all have two types of mesoporous cages, with sizes of 25 Å, 29 Å and 29 Å, 34 Å. The specific surface area is as high as 3100 m^2^/g and 5900 m^2^/g. It can be said that the emergence of these two materials is a milestone in the development history of MOFs. In 2006, Yaghi et al. (Park et al., 2006) synthesized 12 imidazole framework materials with 7 typical topological structures of silica aluminum molecular sieves ZIF-1 to ZIF-12. These materials exhibited superior thermal and chemical stability, among which ZIF-8 and ZIF-11 not only stabilized up to 550°C, but also remained stable in boiling alkaline aqueous solutions and organic solvents. Up to now, MOFs stands out from many other nanomaterials due to its special physicochemical properties and has emerged as one of the most promising nanomaterials for biomedicine. The most common MOFs consist of an infinite lattice formed by metal ions or clusters connected to an organic ligand by strong coordination bonds, and usually exhibit a two or three-dimensional structure (Cao et al., 2021). Due to their unique structures, MOFs have excellent properties for biomedical applications, such as high specific surface area metal (specific surface area in the range of 1,000–10,000 m^2^/g), high porosity, chemical stability, structural tunability, and biocompatibility (Furukawa et al., 2013). MOFs release biologically active metal ions and provide inducers to promote osteogenesis and angiogenesis, deliver immune regulatory signal, and eliminate Reactive oxygen species (ROS) to alleviate inflammation. It can also act as drug carriers, photosensitizers and nano enzymes for antimicrobial and antitumor effects, etc. In this paper, we will review MOFs and by discussing the application of various MOFs composites in common orthopedic diseases, aim to guide and inspire for the treatment of bone diseases as well as the creation of efficient nano materials (Figure 1).

Therefore, this article reviews the recent research of MOFs in bone tumors, osteoporosis and osteoarthritis. In addition to summarizing the previous studies of MOFs in orthopedic diseases, the application potential of MOFs in orthopedic diseases was also discussed.

### 1.1 MOFs in bone tumors

Bone tumors are rare and heterogeneous group of tumors that occur in the skeleton, including many types of malignant and benign tumors, they are one of the major diseases affecting bone health. Among them, osteosarcoma is the most common primary bone tumor and the third most common cancer in the world, it tends to occur more frequently in children and adolescents with rapid bone growth and development. Currently, the treatment of osteosarcoma remains a combination of preoperative neoadjuvant chemotherapy, extensive surgical resection, and postoperative adjuvant chemotherapy. However, even with this treatment modality, tumors recur in 30%–40% of patients and the prognosis for recurrence is poor, with only 23%–29% of patients surviving more than 5 years after second diagnosis (Luetke et al., 2014). Although anticancer drugs can inhibit tumor growth and metastasis throughout the treatment process, chemotherapeutic drugs have the disadvantages of low efficacy, high side effects, and the need for high-dose shock, which leads to huge limitations in the treatment of bone tumor drugs. Therefore, the development of biomaterials with precise and improved anti-tumor capabilities is crucial for bone tumor treatment. MOFs can play a great role by acting as carriers, anti-tumor agents, and drug synergistic systems.

#### 1.1.1 MOFs-mediated chemotherapeutic drug delivery

**FIGURE 2:**
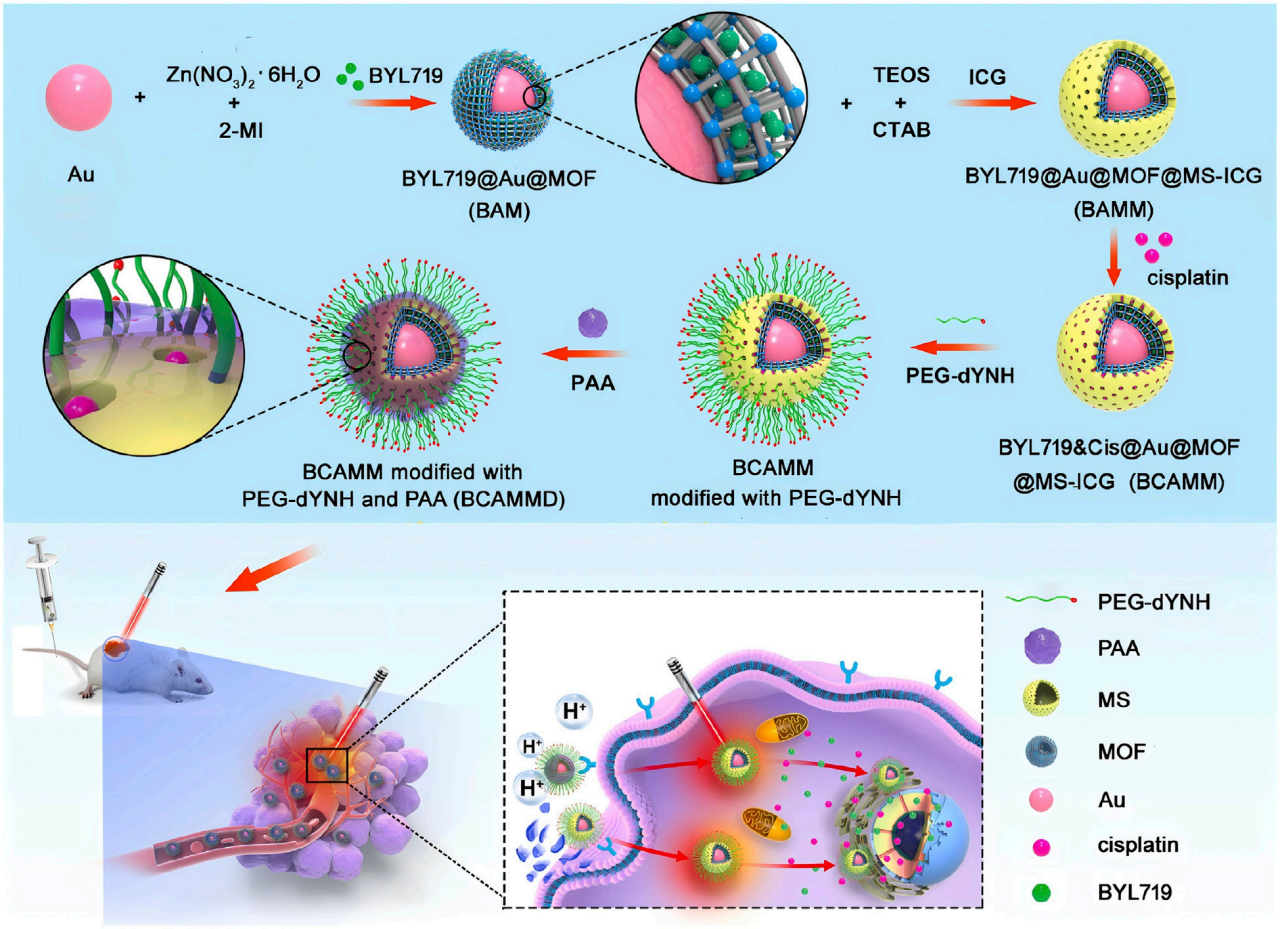
Synthesis process scheme of BYL719 cisplatin bilayer nanoparticles (BYL719&Cisplatin@Au@MOF@MS-ICG, BCAMM) modified dYNH targeting peptide and its antitumor mechanism in tumor cells (Ma et al., 2021). Reproduced under the terms of the Copyright Clearance Center (CCC) license. Copyright ^©^ 2021 Elsevier.

A novel adriamycin drug delivery system (folic acid/metal organic framework/adriamycin) was developed by taking advantage of the high expression of folate receptors on the surface of tumor cells, this system integrate folic acid and MOFs, which significantly improved the specificity and efficiency of adriamycin delivery (Xu et al., 2020). Ma et al. (2021) utilized the MOFs loading of apelalis to form Apelalis@Au@MOFs, and the mesoporous silica structure was used for the successful loading of the photosensitizer indocyanine green and chemotherapeutic drug cisplatin to form apelalis&cisplatin@Au@ metal-organic framework@mesoporous silica-indocyanine green double-shell nanoparticles (Figure 2). The nanoparticles were able to cluster at the spinal tumor under the effect of targeted peptide and high permeability and long retention effect, which resulted in the precise release of Aperis and cisplatin, achieving effective killing of tumor cells and reducing bone destruction caused by tumor metastasis. Wuttke et al. (Luzuriaga et al., 2019) assemble His-tag-functionalized biomolecules with abundant binding sites on the MOFs, the MOFs-based “self-assembled multifunctional ligand particles” that successfully delivered pro-apoptotic peptides and proteins into cancer cells for therapeutic effects. When MOFs are used to load drugs, the content and specific gravity between different substances can be easily adjusted to optimize synergistic effects and substantially improve therapeutic efficacy. For example, Engelke and Wuttke report differentiated experimental results with different ratios of irinotecan/fluorouracil loaded with liposome-encapsulated MIL-88A (LipMIL-88A) (Zhang et al., 2018). From the above article, it can be found that MOFs have shown great potential in the field of drug delivery. By integrating with different targeting molecules, drugs, and functional components, precise targeting of tumor cells can be achieved, improving therapeutic efficacy and reducing side effects.

#### 1.1.2 MOFs as antitumor agents

MOFs can also be prepared from many functional linkers with antitumor effects and metal ions, and these pairings represent the generation of novel antitumor agents called bioMOFs. Binding between phosphate aptamers and Zr-based nanoscale metal-organic backbones (Zr-NMOFs) and the embedding of the photosensitizer TMPyP4 in the structure of G-quadruplex DNA, the preparation of TMPyP4-G4-Aptamer-NMOFs. This nano system induced 90% target cell death and maintained more than 76% inhibition throughout the entire experimental period of this nano system in the treated group (Wang et al., 2019; Meng et al., 2018). Therefore, by designing functional linkers and MOFs reasonably, the treatment system can have better targeting, reduce damage to normal cells, and improve treatment efficacy.

#### 1.1.3 MOFs in tumor photothermal therapy

**FIGURE 3:**
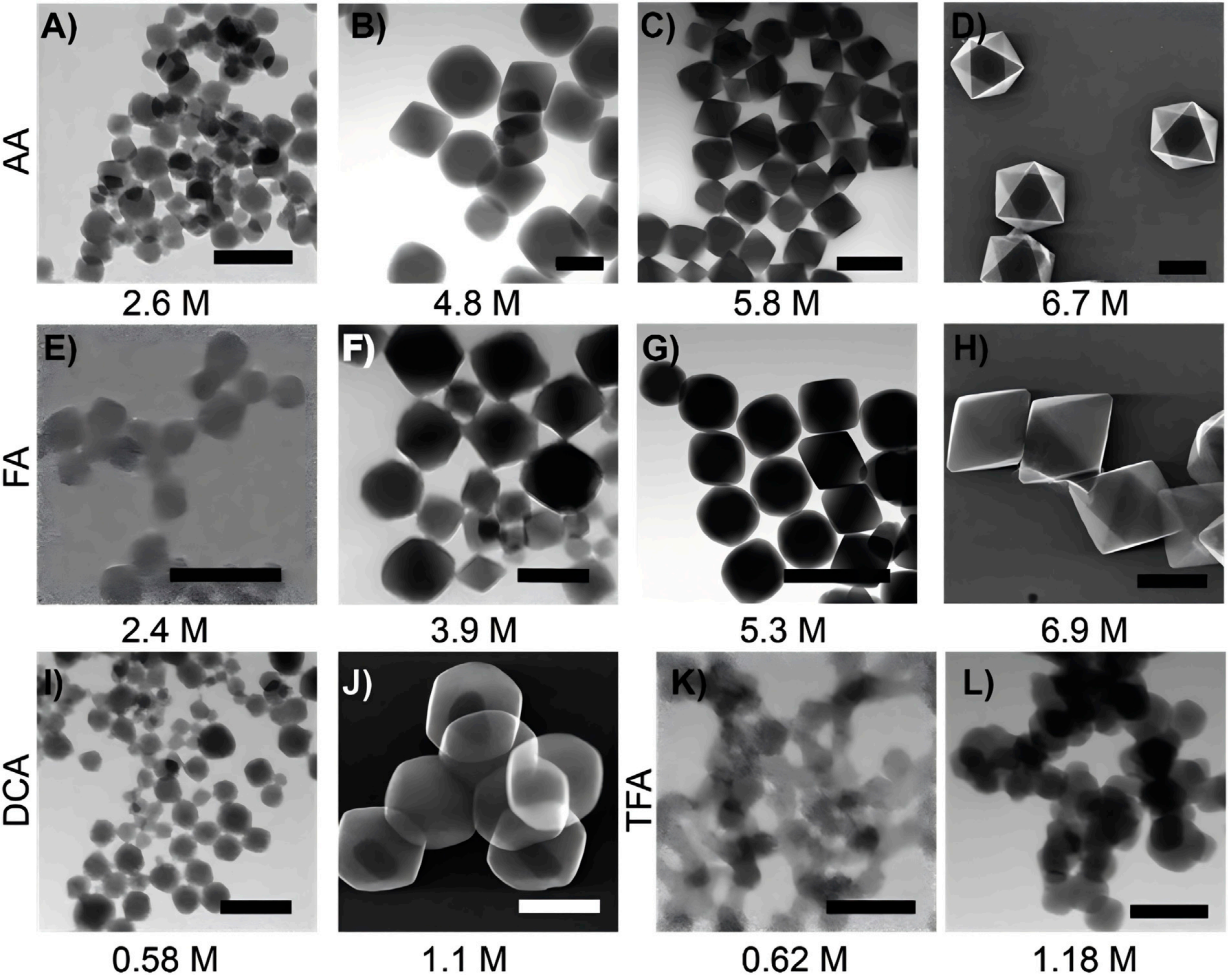
Scanning transmission electron microscopy (STEM) images of UiO-66 synthesized using different acid modulators and concentrations are presented as follows. When synthesized with acetic acid, UiO-66 appears as follows: **(A)** at a concentration of 2.6 M, **(B)** at 4.8 M, **(C)** at 5.8 M, and **(D)** at 6.7 M. When synthesized with formic acid, UiO-66 is as follows **(E)** at 2.4 M, **(F)** at 3.9 M, **(G)** at 5.3 M, and **(H)** at 6.9 M. For UiO-66 synthesized with dichloroacetic acid: **(I)** at 0.58 M and **(J)** at 1.1 M. When synthesized with trifluoroacetic acid, UiO-66 is shown as: **(K)** at 0.62 M and **(L)** at 1.18 M. The scale bars are as follows: A = 100 nm, B = 200 nm, C = 500 nm, D = 500 nm, E = 100 nm, F = 100 nm, G = 1,000 nm, H = 1,000 nm, I = 100 nm, J = 500 nm, K = 100 nm, and L = 500 nm (Morris et al., 2017). Reproduced under the terms of the Copyright Clearance Center (CCC) license. Copyright ^©^ 2017, American Chemical Society.

MOFs with active lymph nodes can be used for chemo dynamic therapy (CDT), radiotherapy (RT), and catalytic modulation of the tumor microenvironment. In addition, MOFs using photosensitizers, chemotherapeutic agents and peptides as active linkers can be used in photo dynamic therapy (PDT), photothermal therapy (PTT) and chemotherapy (Lu et al., 2018). Therefore, it is important to discover MOFs with intrinsic anti-tumor activity and smart connectivity synergistic system to build anti-tumor platforms (Figure 3) (Gong et al., 2020; Morris et al., 2017). PDT has attracted much attention in recent years as an up-and-coming tool for tumor therapy. Using laser and photosensitizers, MOFs attached with active antitumor agents can produce ROS, which can inhibit the development of cancer cells. Organic photosensitizers are mainly involved in type II PDT, it is a process of converting oxygen into toxic singlet oxygen. The photosensitized portion of MOFs is highly ordered and separated, the molecules are prone to diffusion within the porous structure of MOFs, so MOFs-based photosensitizers can generate a large amount of ROS, which can dramatically increase the therapeutic efficacy of PDT (He et al., 2019). Li, et al. (Liu et al., 2020) have constructed a triphenylphosphine (TPP) functionalized up conversion nanoparticle/pyrrole MOF Janus structure (UCMTs) for near-infrared mediated mitochondria-targeted PDT. Under 808 nm near-infrared irradiation, the mitochondria ROS eruption induced by UCMTs can further trigger the intrinsic apoptotic pathway, enhancing the effect of tumor elimination. This nano system enhances the penetration depth of MOFs and the therapeutic effect of photodynamic therapy. Loading the metal organic framework composed of cobalt porphyrin into calcium phosphate bone cement can give it certain photothermal conversion performance, which can target and kill residual tumor cells after osteosarcoma surgery, avoiding tumor recurrence (Deng et al., 2023). DENG et al. (Qu et al., 2021a) developed a core-shell MAZG nanocomposite (mesoporous dopamine/azodiimidazoliumpropane hydrochloride@ZIF-8/Gambogic acid), which was loaded with mesoporous dopamine of azodiimidazoliumpropane hydrochloride in the core. Mesoporous dopamine, and the outer shell consists of ZIF-8 loaded with Gambogic acid, which is encapsulated into the ZIF-8 as an inhibitor specifically targeting heat shock protein 90. The combination of arabinosylcytosine (Ara) and IR820 forms AraIR820, in which the sulfonic acid group of IR820 has a strong interaction with ZIF-8, significantly improving the drug loading efficiency of Ara. After further modification with hyaluronic acid (HA), the final HA/Ara-IR820@ZIF-8 is used for tumor-targeted chemo-photothermal combination therapy, resulting in a tumor inhibition rate of 89%. Therefore, MOFs, as a multifunctional therapeutic platform, combined with various treatment methods such as PTT, CDT and PDT, demonstrate multifaceted intervention capabilities for tumors. This comprehensive treatment strategy can improve treatment effectiveness, reduce side effects, and is expected to become an important direction for future cancer treatment.

#### 1.1.4 MOFs induce ROS production

**FIGURE 4:**
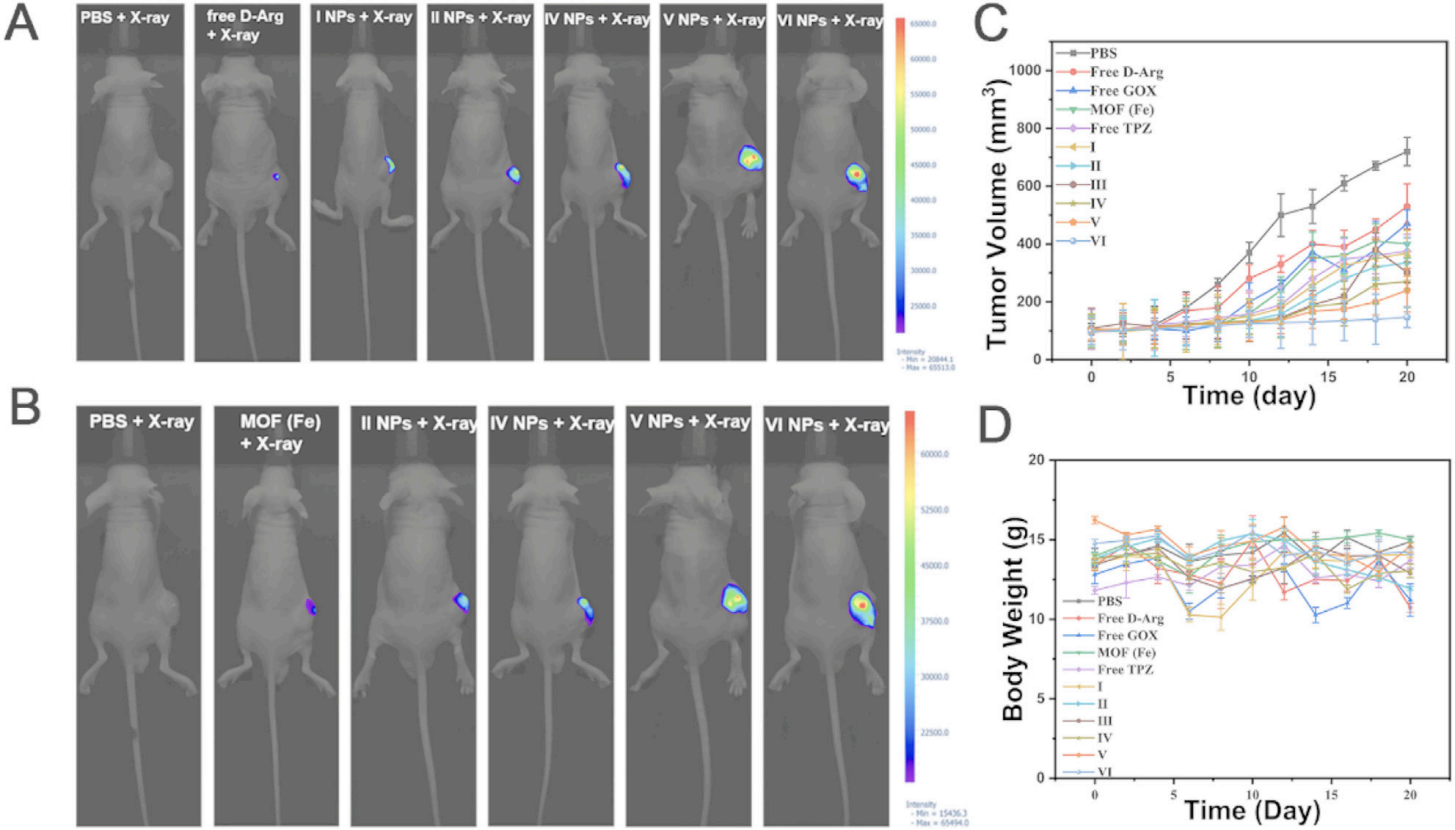
The *in vivo* anti-tumor experiment results for 143b tumor-bearing mice are as follows. **(A)** Fluorescence imaging of NO after X-ray irradiation. **(B)** Fluorescence imaging of ROS after X-ray irradiation. **(C)** The average body weight of mice in the 3 weeks following NP treatments and X-ray irradiation is presented (mean ± SD, n = 5). **(D)** Changes in tumor volume in the 3 weeks following NP treatment and X-ray irradiation are shown (mean ± SD, n = 5). MOF (Fe) loaded with D-Arg (I), MOF (Fe) loaded with D-Arg, GOX (II), MOF (Fe) loaded with D-Arg, GOX and TPZ (III), MOF (Fe) loaded with D-Arg, GOX and TPZ, and cloaked with PDA (IV), MOF (Fe) loaded with D-Arg, GOX and TPZ, cloaked with PDA, and grafted with Fe3+(V),MOF (Fe) loaded with D-Arg, GOX and TPZ,cloaked with PDA, and grafted with Fe3+and FA-BSA (VI) (Wang et al., 2023). Reproduced under the terms of the Copyright Clearance Center (CCC) license. Copyright ^©^ 2023 Elsevier.

PDTDEN et al. (Yuan and Zhang, 2017) design porphyrin-zinc-based MOF (ferrocenylbenzoic acid-ZnTCPP) was constructed using the acoustic sensitizer TCPP, which is capable of releasing ROS upon ultrasonic stimulation, and at the same time ferrocenylbenzoic acid can catalytically convert hydrogen peroxide into oxygen, thus avoiding the problem of oxygen deprivation in the tumor microenvironment that may weaken the effect of acoustic power therapy. Li et al. (2024) modified ZIF-90 with zoledronic acid to enable bone targeting. Then the photosensitizer dihydroporphyrin Ce6 was loaded on it to construct Ce6@ZIF-PEG-ZOL (Ce6@ZPZ), which releases ROS under the irradiation of near-infrared light to kill tumor cells through photodynamics, meanwhile synergistically inhibits tumor growth through the Wahlberg effect with the glycolytic agent 2-deoxy-D-glucose to strengthen the effect of photodynamic therapy. Chemodynamic therapy is an emerging method of tumor treatment, and its working principle is mainly based on the Fenton reaction or Fenton-like reaction, in which transition metal ions (Fe^2+^) convert hydrogen peroxide in the body into ROS to kill cancer cells or inhibit tumor growth. Based on this principle, Wang et al. (2023) constructed a polydopamine-coated iron-based MOF and modified it by a polydopamine coating, combined folic acid bovine serum albumin to achieve the targeting of the tumor site and at the same time function as a contrast agent for magnetic resonance imaging. By loading D-arginine, which can be converted into nitric oxide, glucose oxidase, and block the energy supply of tumor cells, as well as the chemotherapeutic drug tirazamine, a synergistic effect of multiple therapeutic means was achieved (Figure 4). Overall, the understanding and utilization of ROS play a crucial role in tumor therapy. By designing different MOFs systems to regulate changes in ROS, precise killing of tumor cells can be achieved, providing new possibilities for personalized therapy and the implementation of multiple treatment strategies. Future research should further explore the mechanism of ROS in tumor treatment and optimize the design of MOFs to achieve more effective therapeutic effects.

#### 1.1.5 MOFs enhance radiotherapy effects

Radiotherapy is a common method of treating tumors by using ionizing radiation to induce DNA damage, which not only directly damages cancer cells, but also induces the production of reactive oxygen species and the release of tumor-associated antigens to stimulate an immune response, and then induces immunogenic cell death. Osteosarcoma is not sensitive to radiotherapy, and high doses of radiation (up to 80Gy) are often required, which may cause serious adverse effects. MOFs have ultra-small 3D array structure of metal-organic nano framework with loose pores, which is conducive to the scattering of secondary photons and electrons, they can greatly enhance the sensitization effect of high-energy rays. Secondly, the ordered structure of MOFs show large mass attenuation coefficient and a strong dose-enhancing effect, while the pore structure of the nanometallic organic framework can be used to encapsulate drugs or contrast agents for multimodal imaging-guided synergistic oncology therapy. Du et al. (2021) loaded D-arginine into MIL-100(Fe) nanocarriers, under low-dose X-ray irradiation, nitric oxide released from arginine can downregulate hypoxia-inducible factor 1α to alleviate tumor hypoxia, Fe^3+^ catalyzes the reaction of reactive oxygen species produced by hydrogen peroxide with nitric oxide to generate peroxynitrite anion and other reactive nitrogen species. Therefore, hydroxyapatite@metal-organic framework/D-arginine can effectively inhibit tumor growth and prevent recurrence and lung metastasis of osteosarcoma. Li et al. (2023) developed a TZM radiation sensitizer using tantalum (Ta) and zirconium (Zr) as metal nodes and TCPP as photosensitive ligands. Ta-Zr co doping helps to transfer energy to TCPP, thereby generating singlet oxygen and achieving radiodynamic therapy. TZM induces immunogenic cell death and promotes dendritic cell maturation, also upregulates the expression of programmed cell death protein 1 through the cGAS-STING pathway, thereby triggering a powerful anti-tumor immune response. The impact of radiotherapy on patients has always been a difficult problem for doctors to solve. The advantages of MOF systems, such as enhancing radiation therapy efficacy and inducing immune responses, make radiation therapy safer and more efficient during the treatment process, and provide new ideas and possibilities for radiation therapy.

### 1.2 MOFs in osteoporosis

**FIGURE 5:**
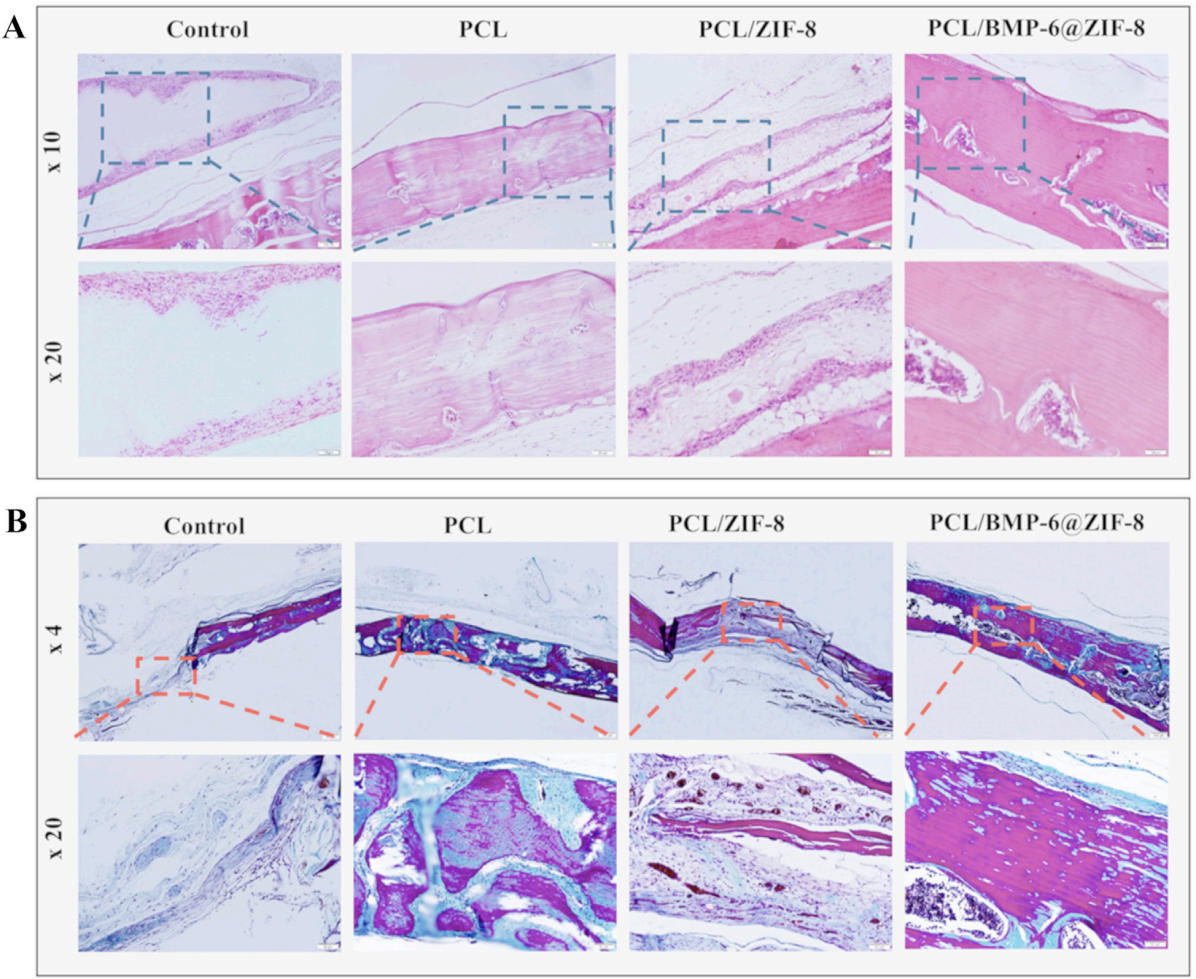
After 8 weeks of implantation, H&E staining **(A)** and Masson’s trichrome staining **(B)** were carried out on the repaired bone defects (Toprak et al., 2021). Reproduced under the terms of the Copyright Clearance Center (CCC) license. Copyright ^©^ 2021 Elsevier.

Osteoporosis is a serious disease characterized by a persistent decrease in bone mass and destruction of bone structure, leading to fragile bones and an increased risk of fractures. Osteoporosis affects 200 million people worldwide, with a higher prevalence in the elderly and postmenopausal women (Strom et al., 2011). The burden on the population of complications associated with osteoporosis such as fractures, bone defects, and lack of bone growth after defects is enormous, including significant morbidity, reduced quality of life, increased mortality, and substantial healthcare expenditures (Brown et al., 2021; Marrinan et al., 2015; Bliuc et al., 2009). The adult skeleton undergoes bone remodeling to maintain mineral homeostasis and bone quality (Armas and Recker, 2012; Farlay et al., 2019). Bone remodeling consists of resorption by multi-nucleated, myeloid-derived osteoclasts (OCs) followed by formation by mesenchymal-derived osteoblasts (OBs). This coordination of bone remodeling activities is commonly referred to as the coupling of bone resorption to bone formation. With aging and other conditions, such as gonadal hormone loss, the rate of resorption exceeds formation resulting in reduced bone mass (Sims and Martin, 2020). In other pathological conditions, resorption occurs independently from subsequent bone formation leading to destructive bone loss (Feng and Mcdonald, 2011). This can result in a reduction in bone mass, deterioration of the microstructure, and an elevated risk of fragility MOFs are able to stimulate the activity of bone mesenchymal stem cells and promote the process of new bone formation and mineralization. Studies have shown that MOFs play a key role in promoting osteoblast growth, inhibiting osteoclasts, and facilitating angiogenesis and bone mineralization, providing novel strategies and ideas for the effective treatment of osteoporosis (Matlinska et al., 2019; Zheng et al., 2020; Dang et al., 2020; Qu et al., 2021b; Dong et al., 2023; Pan et al., 2023; Lin et al., 2024) (Figure 5) (Toprak et al., 2021).

#### 1.2.1 MOFs promote osteogenic gene expression

Proliferation and differentiation of osteoblasts play a key determinant role in the bone repair process. During the early stages of osteogenesis, MOFs promote the osteogenic process by regulating the transcriptional activity of Runx2, a gene central to the osteoblast phenotype (Wang et al., 2024). It was demonstrated that Sr^2+^ loaded on copper 1,3,5- phenylenetricarboxylic acid significantly increased the transcriptional activity and phosphorylation of Runx2, and that a variety of MOFs were effective in promoting the expression of the Runx2 gene (Farasati et al., 2023). Ma et al. (2024) designed alendronate (ALN)-mediated defective MOF sonosensitizer, which can effectively clear Methicillin-resistant *Staphylococcus aureus* (MRSA) infections and promote osteogenic differentiation under differential ultrasonic irradiation. In the presence of zirconium–phosphate coordination, the ALN-mediated porphyrin-based MOF (HN25) with a proper defect has great sonodynamic antibacterial efficiency (98.97%, 15 min) and bone targeting ability. Ren et al. (2022) encapsulation of the SCM in ZIF-8 nanoparticles, which enhances osteogenic fractionation of MSCs and accelerates bone regeneration *in vivo*. The targeting of MOFs nanoparticles represented by SCM/ZIF-8 was demonstrated and their regulatory role on MSCs was confirmed. Many genes associated with osteogenic *in vivo* differentiation were observed to be upregulated in MSC cultures treated with SCM/ZIF-8, with involvement in the cAMP signal pathway. The efficacy of SCM/ZIF-8 nanoparticles in enhancing bone regeneration was demonstrated in a bone defect model through a series of rigorous experimental procedures. Some researchers found that by utilizing type I collagen, which has its own mechanical support ability and promoting cell attachment, a multifunctional and biodegradable scaffold system was constructed by combining collagen scaffolds with interleukin 4-metal-organic framework @CaP, which provided multiple osteogenic nucleation sites for the bone regeneration multicellular unit and realized functional bone regeneration *in vivo* (Zheng et al., 2020). In the later stages of osteogenesis, bone bridging eggs function by interfering with cell adhesion and migration, while osteocalcin can bind to Ca^2+^, regulate calcium ion homeostasis, and participate in the bone mineralization process. On the other hand, MOFs increase the expression levels of these proteins through transcription and translation processes, thus promoting osteogenesis (Wang et al., 2023). Chen et al. (2024) successfully prepared bimetallic Mg/Cu-MOF coatings on pure Zn, with the intention of promoting osteogenesis, angiogenesis and antimicrobial activity of Zn substrates. The findings demonstrated that the degradation rate and water stability of the Mg/Cu-MOF coatings could be controlled by adjusting the proportion of Cu. This significantly enhanced modulated the inflammatory response and promoted the vascularization of endothelial cells, while exhibiting excellent antimicrobial activity. Therefore, the high surface area and pore structure of MOFs can be used for effective loading of drugs or growth factors, which can help promote bone cell proliferation and differentiation. They can also be used for anti-inflammatory, antibacterial and other functions, which can help maintain the healthy state of bone tissue.

#### 1.2.2 Osteoclast inhibition by MOFs

A decrease in osteoblasts or an increase in osteoclasts disrupts the structure of bone tissue, greatly increasing the risk of osteoporosis as well as disorders such as osteogenesis imperfecta. Osteoprotegerins are proteins that play a key role in bone metabolism by binding to the ligand of the nuclear transcription factor κB receptor (RANKL) activator, thereby inhibiting osteoclast formation and activity. Antitartrate acid phosphatase is a signature indicator representing osteoclast activity. Therefore, the process of bone resorption can be effectively inhibited by increasing the secretion of osteoprotegerin and decreasing the secretion of anti-tartaric acid phosphatase. The synthesis of a bimetallic metal-organic skeleton (Pt@ZIF-8@La) was achieved by encapsulating metallic platinum (Pt), which exhibits excellent physicochemical and biological properties, in ZIF-8. This was followed by the introduction of lanthanum (La), an osteogenic active element, through ion exchange. This nanoplatform exhibits the functions of efficient ROS scavenging, immunomodulation and promotion of osteogenic differentiation. In addition, Pt@ZIF-8@La has been demonstrated to promote osteogenic mineralization by up-regulating the ratio of osteoprotegerin (OPG)/receptor activator of NF-κB ligand (RANKL). This leads to a synergistic therapeutic effect of immunomodulation, osteogenesis, and alleviates aseptic osteolysis (Pan et al., 2023). Zoledronic acid-functionalized MOFs can be used to combat rheumatoid osteoclasts in rheumatoid arthritis. Osteoclast hyperactivation in arthritis. The ability to block osteoclast binding to bone matrix, thereby interfering with the nuclear transcription factor κB receptor activator ligand/nuclear transcription factor κ receptor activator signaling pathway, inhibits osteoclast differentiation and induces apoptosis of osteoclasts in order to counteract osteoclast-mediated bone resorption (Tao et al., 2023). Pang et al. (2020) design surface modification with the anti-osteoclastic bisphosphonate, zoledronic acid (ZOL), to enable bone-targeted immunostimulatory capabilities cytosine-phosphoguanosine (CpG)-loaded MOF. Functionalized bone-targeted immunostimulatory MOF (BT-isMOF) nanoparticles exhibited strong binding to calcium phosphate and demonstrated specific accumulation in bone tissue *in vivo*. BT-isMOF nanoparticles effectively inhibited osteoclast formation and induced macrophage polarization towards an M1 pro-inflammatory phenotype. Tao et al. (2023) developed ZIF-8@CRIg-CD59@HA@ZA, its achieved bone-targeted delivery and pH-responsive slow release of the complement inhibitor CRIg-CD59. Its surface-mineralized zoledronic acid (ZA) targets the acidic microenvironment of bone in RA, and sustained release of CRIg-CD59 recognizes and prevents the formation of complement membrane attack complexes (MACs) on the surface of healthy cells and significantly inhibits osteoclast-mediated bone resorption. Bone malignancies often cause severe osteoclastic conditions in patients, which are difficult to control or even reverse with conventional treatments. Zou et al. (2022) constructed ICG@Cu2-XSe-ZIF-8 utilized an acidic microenvironment, which allowed ZIF-8 to be cleaved and released large amounts of Cu2-XSe, which could subsequently be degraded to Cu^+^ and Cu^2+^, triggering a Fenton reaction that induced CDT. What’s more, selenium in Cu 2-X Se can regulate selenoproteins and inhibit the production of tumor cells and osteoclasts to reduce the erosion of bone tissue. Moreover, PTT-induced hyperthermia can further enhance the CDT effect in tumors to achieve the synergistic effect of PTT/CDT. Lin et al. (2024) developed an acid-responsive neutralization system with *in vivo* gene editing capability by loading sodium bicarbonate (NaHCO_3_) and RANKL-CRISPR/Cas9 (RC) plasmid into MOF. It was demonstrated that ZIF8-NaHCO3@Cas9 (ZNC) effectively neutralized the acidic microenvironment and inhibited ROS production. The results demonstrated that nanoparticles loaded with NaHCO_3_ exhibited a higher transfection efficiency in an acidic environment compared to nanoparticles without this loading. Protection by bi-directionally promoting osteogenesis and inhibiting osteolysis. It takes 3 months for osteoblasts to form new bone, while osteoclasts only take 3 weeks to break a bone fracture. Therefore, in correcting the dynamic balance between osteogenesis and osteoclastogenesis in osteoporosis, inhibition of osteoclastogenesis is particularly crucial. From the above content, it can be concluded that MOFs can effectively inhibit bone resorption, thereby promoting bone tissue regeneration and repair. Therefore, when facing osteoporosis, more attention should be paid to this aspect.

#### 1.2.3 MOFs for angiogenesis

Kusumbe et al. (2014) discovered that capillaries can be classified into H-type and L-type. The number of H-type vessels can be used as a diagnostic indicator of the state of vascular growth and osteogenic capacity, influencing bone metabolism in many ways, including angiogenesis and abundance. Mi et al. (Leroux et al., 2020) demonstrated that electrical stimulation of the dorsal root ganglion (DRG) at L3 and L4 in rats resulted in the activation of the Ca/CaMKII/CREB signal pathway and action potentials, which directly promoted CGRP synthesis and release and induced osteoporotic fracture healing and H-vessel formation. This breaks the traditional “triadic theory” of bone metabolism and updates it to a new quadratic theory of “peripheral neuroangiogenesis - Osteoclasts - osteogenesis”. Kang et al. (2022) utilized human adipose-derived stem cells (hADSCs-exos), Mg and GaMII/CREB signal pathway and action potentials, which directly promoted CGRP synthesis and release and induced osteoporotic fracture healing and H-vessel formation. Using hADSCs-exos, Mg and GA, a unique exosome-functionalized free cell scaffold (PLGA/Exo-Mg-GA MOF) was designed and synthesized. This nano scaffold has the functions of stabilizing bone grafting environment, promoting osteogenic differentiation, angiogenesis and anti-inflammatory ability, and accelerating bone reconstruction. Dang et al. (2020) successfully prepared a nanosheet-structured β-tricalcium phosphate (TCP) (Cu-TCPP-TCP) scaffold by using 3D printing technology in combination with an insitu growth method in a solvothermal system. The excellent photothermal effect of Cu-TCPP nanosheets allows for the modulation of photothermal performance through changes in nanosheet content, ambient humidity, and near-infrared light power density. The nano scaffold was found to be capable of supporting the attachment of human bone marrow stromal cells (HBMSCs) and human umbilical vein endothelial cells (HUVECs). This resulted in the promotion of bone generation in all aspects. The angiogenesis-related genes VE-cadherin and endothelial-type nitric oxide synthase are key vascularization. Qu et al. (2021b) prepared a cobalt-based organometallic framework (Co-TCPP)-modified calcium phosphate bone cement using Co and (4-carboxyphenyl)porphyrin (TCPP), and verified the great potential of Co-TCPP-calcium phosphate bone cement in the treatment of tumor-borne bone defects by up-regulating the content of angiogenesis-related genes, VE-cadherin and endothelial nitric oxide synthase, and by promoting the blood vessel regeneration. In addition, Cu^2+^ also possesses pro-angiogenic activity, while Mg^2+^ has been shown to activate hypoxia-inducible factor 1a after influx into the cell via magnesium transporter protein 1, which stimulates the transcription of vascular endothelial growth factor and induce angiogenesis (Xiang et al., 2023; Xu et al., 2023; Xiao et al., 2021). In addition to the three metal-centered MOFs mentioned above, others can also be used as carriers for loading pro-angiogenic small molecule drugs to promote angiogenesis (Gutowska et al., 2005). These studies reveal the potential of MOFs in promoting angiogenesis and bone tissue regeneration. By regulating the generation of blood vessels and activating signaling pathways, MOFs can affect bone metabolism and promote fracture healing and bone reconstruction processes.

#### 1.2.4 MOFs induced bone mineralization

Bone mineralization is the process by which bone minerals, such as hydroxyapatite, precipitate on an organic collagen scaffold to eventually form a bone matrix. Bone mineralization provides the necessary support and stability for newborn bone tissue and is a critical step in bone repair. Metal ions in MOFs, such as Mg^2+^, Zn^2+^, Mn^2+^, Sr^2+^, etc., can affect the composition of hydroxyapatite by ion exchange, at the same time, changes in bond lengths induced by ion exchange can lead to deformation of the crystal structure of hydroxyapatite, which in turn affects the process of bone mineralization. In addition, Sr^2+^ has a high affinity for bone tissue, and the Sr-hydroxyapatite-metal-organic framework composite coatings constructed by Zhang et al. (2019). The significantly enhance the early osteogenesis and the integration of bone implants. Meanwhile it projected a good result in alkaline phosphatase activity and bone mineralization. In addition, some MOFs have metal ions with ionic radii close to those of Ca^2+^, which can also promote phosphate deposition through ion exchange, such as La^3+^, and it has been demonstrated that the interfacial bonding strength of La-hydroxyapatite coatings is significantly enhanced after doping with La (Lou et al., 2015). From the above content, it can be concluded that metal ions in MOFs play an important role in the process of bone mineralization. It can affect the formation and stability of new bone tissue by regulating ion exchange and crystal structure. This provides new ideas and possibilities for designing and developing new bone repair materials, which are expected to play an important role in fields such as fracture healing and bone defect repair.

### 1.3 MOFs in osteoarthritis

Osteoarthritis (OA) is estimated to affect 300 million or more people worldwide and is now the leading cause of disability in the elderly. It is often associated with chronic pain, progressive loss of function and a significant reduction in quality of life (James et al., 2018; Cisternas et al., 2016; Nguyen et al., 2014; Hunter et al., 2014; Centers for Disease Control and Prevention, 1995). OA was thought to be a simple degenerative disease characterized by chronic overload and biomechanical impairment of the joints, leading to destruction of articular cartilage and resulting inflammation, followed by stiffness, swelling and loss of function. However, with the deepening of research, scholars have concluded that OA is an extremely complex pathological process composed of inflammatory and metabolic factors. Factors such as trauma, infection, obesity, genetics, metabolic syndrome, atherosclerosis, endocrine system and estrogen abnormalities, and aging (chondrocyte senescence, DNA damage, cartilage matrix aging, oxidative stress, mitochondrial dysfunction, and autophagy) can influence the development of OA (Berenbaum, 2013; Loeser, 2011; Yusuf et al., 2010). Due to the complexity and serious impact of OA, exploring new treatment methods and materials has become an urgent task. MOFs has shown remarkable potential in the treatment of osteoarthritis due to its unique structure and properties.

#### 1.3.1 MOFs for immunomodulation

**FIGURE 6:**
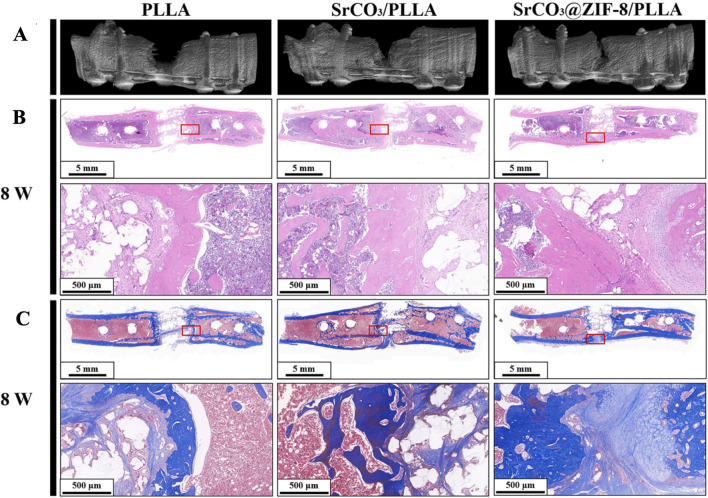
After 8 weeks of implanting PLLA, SrCO₃/PLLA, and SrCO₃@ZIF-8/PLLA scaffolds, the three-dimensional reconstruction images **(A)**, H&E staining **(B)**, and Masson trichrome staining **(C)** of femoral defects are shown (Qian et al., 2024). Reproduced under the terms of the Copyright Clearance Center (CCC) license. Copyright ^©^ 2024 Elsevier.

Macrophages, as key regulators of the inflammatory response and important components of the innate immune system, can be divided into two M1 and M2 subtypes, depending on their function. In the early stages of the inflammatory response, M1 macrophages destroy pathogens by enhancing immune activity, but this pro-inflammatory response inhibits tissue regeneration if it persists for too long. As inflammation progresses, M1-type macrophages convert to M2-type and secrete anti-inflammatory cytokines such as interleukin 4, interleukin 10, and arginase 1 to alleviate excessive inflammation. Qian et al. (2024) devised a core-shell structure (SrCO_3_@ZIF-8) that generates ZIF-8 *in-situ* on the surface of SrCO_3_, and utilized an organic ligand for ZIF-8 with poly (L-lactide) to form a structurally robust composite scaffold (Figure 6). The scaffold not only exhibited excellent mechanical properties, but also promoted the conversion of M0-type macrophages to M2-type macrophages by releasing Sr^2+^ and Zn^2^⁺, decreased the expression of tumor necrosis factor, and increased the expression levels of interleukin 10 and arginase1, thereby optimizing the repair microenvironment and significantly promoting the osteogenic differentiation of mouse MSCs. Chen et al. (2022) utilized the immunomodulatory effect of AHT-Ce/Sr metal-organic framework to achieve a decrease in the expression of interleukin 1β and tumor necrosis factor α and an increase in the M2 macrophage-associated marker CD206 in macrophages. Yan et al. (2022) constructed fluorine-doped multifunctional zirconium-based metal-organic skeleton thin films on titanium-based implants. The fluorine-doped zirconium-based metal-organic skeleton stimulated the release of fumaric acid and induced the polarization of macrophages to M2 type. Rheumatoid arthritis (RA), an important component of OA, often exhibits more severe clinical symptoms and rate of progression. Inflammatory infiltration and bone destruction are important pathological features of RA originating from a disturbed ecological niche of macrophages. RA is mediated by the disruption of the barrier function of VSIg4 lined macrophages due to hyperactivation of complement mediating the inflammatory infiltration within the joint. Tao et al. (2023) designed the ZIF8@CRIg-CD59@HA@ZA nanoplatform to address this issue. This recognized and blocked complement membrane attack complex (MAC) on the healthy cell surfaces, reducing the deposition of complement markers and osteoclast inhibition. Moreover, this nanoplatform facilitated the regeneration of VSIg4 synovial macrophages. The successful application of this dual-targeted therapeutic vector indicates that the restoration of the synovial macrophage ecological niche represents an effective therapeutic strategy for RA. So, it can be inferred that MOFs have ability to regulate macrophage activity, inhibit inflammatory factors, promote the release of anti-inflammatory factors, and optimize the repair of the inflammatory microenvironment.

#### 1.3.2 Targeted delivery of anti-inflammatory drugs by MOFs

In terms of arthritis drug therapy, clinical choices are mostly non-steroidal anti-inflammatory drugs (NSAIDs), glucocorticoid, anti-rheumatic drugs (DMARDs), and biologics that control pain and inflammation (Feng and Chen, 2018). However, defects such as poor water solubility, uncontrolled drug release, low cell permeability and early degradation before reaching the target site, and undesirable side effects significantly limit the bioavailability of drugs (Majumder and Minko, 2021). MOFs, as highly efficient drug carriers, have the advantage of being able to accurately control the release of drugs, thanks to the fact that MOFs, in response to signals from an external stimulus, change their own topology. This is mainly because of when the MOFs receives the external stimulus response signal, its topology, size and pore diameter will change, thus realizing the precise control of drug release rate. The large specific surface area of MOFs and the activation and protection of drugs can not only significantly improve the therapeutic efficiency, but also significantly reduce the systemic adverse reactions. For example, loading ketoprofen into Mg metal organic framework significantly reduced the expression of various pain related genes such as cyclooxygenase, significantly upregulated osteoblasts, and downregulated the secretion of pro-inflammatory factors (Ge et al., 2021). Jiang et al. (2023) use ZIF-8 to deliver Neobavaisoflavone (NBIFs), NBIF@ZIF-8 complexes with anti-inflammatory effects were constructed. In addition, MOFs can not only be used as carriers to carry anti-inflammatory drugs, but also improve the therapeutic efficacy of drugs through on-demand design. Xue et al. (2021) developed a dual drug delivery nanoplatform based on MOFs loaded with rapamycin and bilirubin-mesoporous dobutamine, and at the same time, this system was affixed with a collagen II-targeting peptide on its surface, which resulted in a significant enhancement of the targeting to the cartilage. We can see the potential of MOFs as efficient drug carriers in the treatment of arthritis. MOFs can precisely control drug release, improve therapeutic efficacy and reduce adverse reactions. These nano delivery systems are expected to greatly improve the limitations of existing arthritis drug treatments.

#### 1.3.3 Scavenging of pro-inflammatory cytokines and ROS by MOFs

**FIGURE 7:**
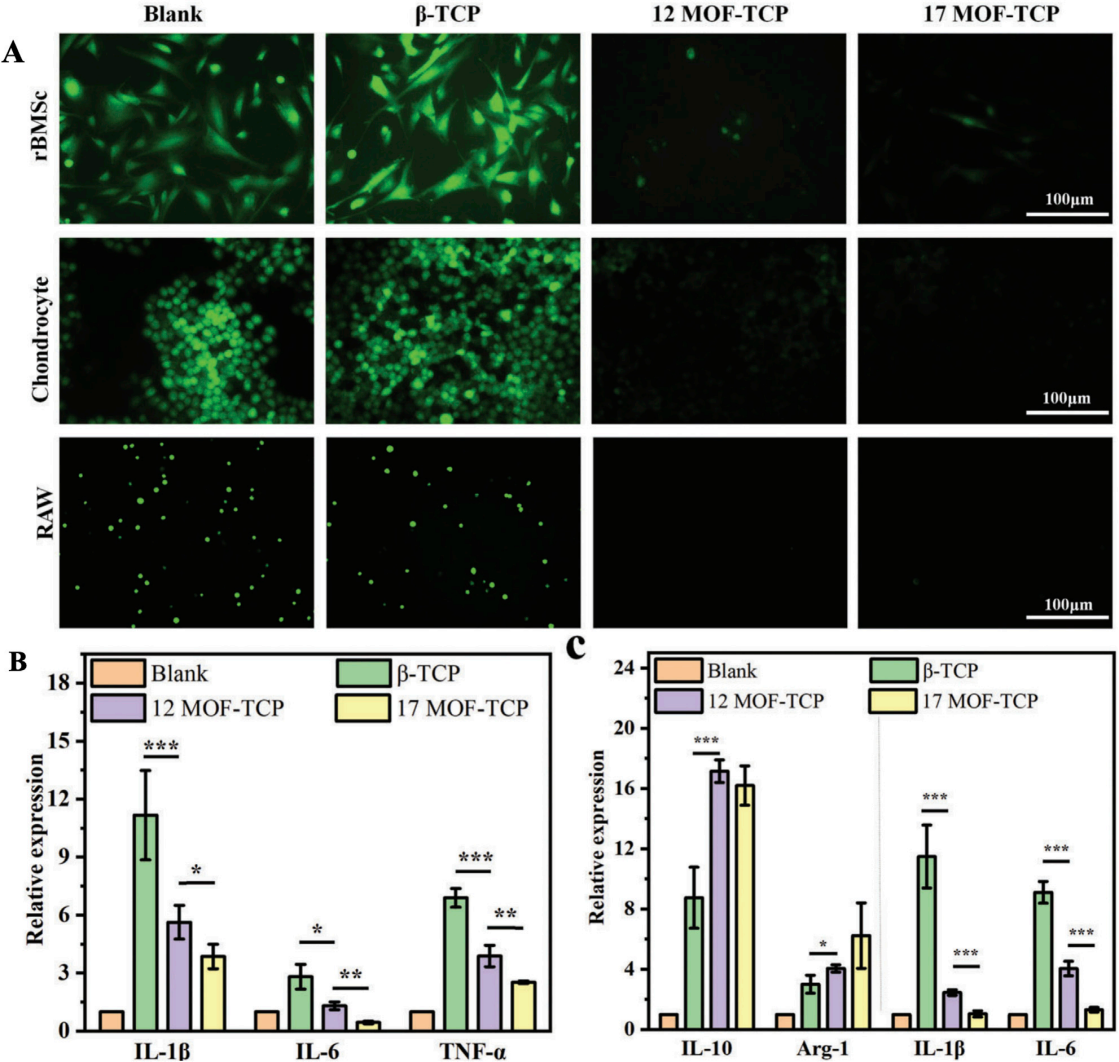
The MOF-TCP scaffolds exhibit *in vitro* antioxidative and anti-inflammatory activities. **(A)** The ROS fluorescence staining images are presented for rBMSCs, chondrocytes, and RAW 264.7 cells cultured with scaffolds under H₂O₂ stimulation. **(B)** The expression of proinflammatory genes in chondrocytes is shown (n = 3). **(C)** The expression of anti-inflammatory genes and proinflammatory genes in RAW 264.7 cells. *p < 0.05, **p < 0.01, ***p < 0.001 (Shu et al., 2023). Reproduced under the terms of the Copyright Clearance Center (CCC) license. Copyright ^©^ 2023 The Authors. Advanced Science published by Wiley‐VCH GmbH. Open access article.

The main pathological feature of osteoarthritis is the accumulation of excessive pro-inflammatory cytokines and reactive oxygen species at the joints, which can exacerbate bone defects and hinder the regeneration of bone and cartilage tissues. Therefore, the removal of pro-inflammatory factors is a direct and effective therapeutic strategy for osteoarthritis. Shu et al. (2023) has successfully developed a multi-functional scaffold, MOF-TCP, with good biocompatibility and modulation of inflammation by functionalizing βTCP scaffolds with Zn/Co-MOF. This nano scaffold can simultaneously enhance the differentiation of rBMSCs and the maturation of chondrocytes by eliminating ROS-mediated inflammatory microenvironment and protect them from oxidative stress, resulting in good regenerative capacity and regenerative environment for osteochondral bone (Figure 7). The exosome functionalized polylactic acid hydroxyacetic acid copolymer/Mg gallic acid metal organic framework scaffold prepared by Kang et al. (2022). This system significantly reduced the levels of pro-inflammatory cytokine induced nitric oxide synthase, cyclooxygenase-2, and reactive oxygen species by releasing exosomes and gallic acid, effectively suppressing harmful immune responses during osteogenesis. HIF-2α plays a catabolic role in OA, and an increase in HIF-2α exacerbated the chondrocyte response to hypoxia and the expression of catabolic factors, cytokines, chemokines, and MMPs in the synovium (Zhang et al., 2015; Pi et al., 2015; Yang et al., 2010). Since HIF-2α has been shown to be a key pathogenic target during OA progression, downregulation of HIF-2 α expression in locally inflamed joints is important for the treatment of OA cartilage (Pasupneti et al., 2020; Yang et al., 2019). Zhang et al. (2023) constructed an injectable drug co-delivery system by loading MIL-101-NH with Curcumin (CCM) and anti-HIF-2α siRNA (siHIF-2α). In an acidic microenvironment, the release of CCM and siHIF-2α from this scaffold has the potential to alleviate the progression of HIF-2α gene silencing in chondrocytes. This is achieved by a synergistic downregulation of OA-associated catabolic markers and an upregulation of cartilage-specific markers in chondrocytes, resulting in a merit therapeutic effect. The above research demonstrates a novel treatment strategy for osteoarthritis. By using MOFs systems to regulate inflammation, promote cell differentiation and suppress harmful immune responses, these novel scaffolds provide new possibilities for improving treatment outcomes in patients with osteoarthritis. The clearance of pro-inflammatory factors and reactive oxygen species, as well as treatment strategies targeting HIF-2 α, are expected to become important means of treating osteoarthritis in the future.

## 2 Conclusion and prospect

MOFs exhibit a number of unique advantages compared to other nanomaterials, such as their high specific surface area and drug loading efficiency. This means that MOFs can accommodate more drug molecules in their structure, which improves therapeutic effectiveness. In addition, MOFs exhibit excellent chemical stability, meaning that they are able to maintain their structural and functional integrity in different chemical environments. MOFs also perform well in terms of biosafety, as they do not cause adverse reactions in living organisms, which is essential for clinical applications. Another notable feature is the pH responsiveness of MOFs, which are able to change their structure or release drugs under different pH conditions, enabling precision therapy. In addition to the above advantages, MOFs can also be combined with a variety of other substances to participate in the diagnostic or therapeutic process. For example, they can be combined with specific gases such as hydrogen and nitric oxide to treat the corresponding diseases. These gases can play an important role in the treatment process, such as anti-inflammatory, antioxidant, etc. In addition, MOFs can be combined with gene therapy techniques to serve as high-capacity vectors for the delivery of specific gene segments. This combination opens up new possibilities for gene therapy, allowing gene fragments to be delivered more efficiently into target cells.

Although much has been summarized in the manuscript about the application of MOFs to orthopedic diseases, in reality, the application of MOFs extend far beyond orthopedic diseases. In the medical field, the application prospects of MOFs are very broad, including but not limited to drug delivery, biological imaging, cancer treatment, antibacterial applications, etc. However, MOFs face some challenges before they can be used in the clinic. Researchers need to establish standardized biosafety evaluation criteria to ensure the safety and effectiveness of MOFs *in vivo*. In addition, safety evaluation in animals is needed to further verify the feasibility of its clinical application. In order for MOFs to be widely used in the clinic, researchers also need to develop new methods for making and storing MOFs. These methods should not only reduce production costs, but also ensure the stability and effectiveness of MOFs in clinical applications. In addition, broadening the application scenarios of MOF is also the part we need to focus on. In the future, we should continue to optimize and improve the physical structure and chemical properties of MOFs to achieve its comprehensive application in more fields. Through these efforts, MOFs are expected to become an important nanomaterial in the future, bringing revolutionary changes to the field of medicine.
